# Machine learning prediction of mortality in Acute Myocardial Infarction

**DOI:** 10.1186/s12911-023-02168-6

**Published:** 2023-04-18

**Authors:** Mariana Oliveira, Joana Seringa, Fausto José Pinto, Roberto Henriques, Teresa Magalhães

**Affiliations:** 1 NOVA National School of Public Health, Universidade NOVA Lisboa, Lisbon, Portugal; 2 Serviço de Cardiologia, Centro Hospitalar Universitário de Lisboa Norte (CHULN), CAML, CCUL, Faculdade de Medicina, Universidade de Lisboa, Lisbon, Portugal; 3 NOVA Information Management School (NOVA IMS), Universidade Nova de Lisboa, 1070-312 Lisbon, Portugal; 4 NOVA National School of Public Health, Public Health Research Centre, Comprehensive Health Research Center, CHRC, Nova University of Lisbon, Lisbon, Portugal

**Keywords:** Machine learning, Cardiovascular diseases, Acute Myocardial Infarction, Predictive models

## Abstract

**Background:**

Acute Myocardial Infarction (AMI) is the leading cause of death in Portugal and globally. The present investigation created a model based on machine learning for predictive analysis of mortality in patients with AMI upon admission, using different variables to analyse their impact on predictive models.

**Methods:**

Three experiments were built for mortality in AMI in a Portuguese hospital between 2013 and 2015 using various machine learning techniques. The three experiments differed in the number and type of variables used. We used a discharged patients’ episodes database, including administrative data, laboratory data, and cardiac and physiologic test results, whose primary diagnosis was AMI.

**Results:**

Results show that for Experiment 1, Stochastic Gradient Descent was more suitable than the other classification models, with a classification accuracy of 80%, a recall of 77%, and a discriminatory capacity with an AUC of 79%. Adding new variables to the models increased AUC in Experiment 2 to 81% for the Support Vector Machine method. In Experiment 3, we obtained an AUC, in Stochastic Gradient Descent, of 88% and a recall of 80%. These results were obtained when applying feature selection and the SMOTE technique to overcome imbalanced data.

**Conclusions:**

Our results show that the introduction of new variables, namely laboratory data, impacts the performance of the methods, reinforcing the premise that no single approach is adapted to all situations regarding AMI mortality prediction. Instead, they must be selected, considering the context and the information available. Integrating Artificial Intelligence (AI) and machine learning with clinical decision-making can transform care, making clinical practice more efficient, faster, personalised, and effective. AI emerges as an alternative to traditional models since it has the potential to explore large amounts of information automatically and systematically.

## Background

Cardiovascular diseases are the leading cause of death in the European Union (EU) and the United States of America, representing approximately 30% of deaths [1–3]. In cardiovascular diseases, Acute Myocardial Infarction (AMI) is still the leading cause of death and hospitalisation in Portugal and globally [3], representing 3.3% of the total deaths in Portugal [4]. Moreover, the effects of COVID‐19 demonstrated the need to maintain access to high-quality acute care for AMI, as significant rises in AMI mortality rates were seen during this period [3, 5, 6]. In recent decades, introducing new technologies, optimising therapeutic means, and preventive policies, improving pre-hospital care, and creating guidelines have substantially impacted the mortality rate and length of hospital stay [3, 7].

However, the 30-day in-hospital AMI mortality rate that reflects the provision of care and clinical interventions [8, 9] significantly varies among EU countries. The lowest rates are found in The Netherlands, Sweden, Slovenia, Denmark, Poland and Ireland, with values below 5.0%. Portugal stands at 7.3%, above the average for EU countries, with an observed increase in 2020 [3].

But, many factors can influence AMI results. According to the European Society of Cardiology, AMI mortality is influenced by several risk factors with significant predictive power in the risk of death, such as age and sex, comorbidities, and high heart rate, but also changes in some laboratory findings [10, 11]. These factors can be managed during the medical emergency, where half of the deaths occur in the first hours after the onset of symptoms, and through early identification, which may help prevent or delay the condition and even prevent death [12–14].

In the digital health era, where we can access thousands of data, ML and data mining algorithms can contribute to clinical decision support [15]. Some examples are early screening and diagnosis, disease prevention that identifies risk factors [16], treatment management and monitoring with improved pharmacovigilance and patient safety, and improved outcomes and care provided [17]. Particularly in cardiovascular diseases [13, 18, 19].

Since cardiovascular diseases are complex and heterogeneous, resulting from genetic, environmental, and behavioural factors [10, 11], there is a growing need to analyse data from different sources of information, namely administrative, laboratory, and imaging, for interpretation, diagnosis, and decision-making. Furthermore, data analysis can reduce waste by optimising resources and installed capacity, improving the patient journey, and interacting with healthcare organisations [20–22] empowered by sophisticated technology.

In recent years, research on ML in AMI has mainly focused on predicting patient mortality [18, 23–28], prediction of patient readmission [29], or the occurrence of arrhythmia after acute myocardial infarction [25] and has already proved to be better predictors than the traditional statistical models [26–28]. Also, we found better performances of ML models than the traditional models, working on AMI mortality analysis in different settings and populations: Europe, the United States and Asia [18, 23–28], mainly predicting one year or 30 days survival after AMI.

In this study, we aim to build a model for predicting mortality in patients with AMI at hospital admission. We propose to measure the impact of introducing cardiac test results and physiological results in addition to administrative data based on machine learning (ML). We introduce in this work the capability of the model mortality prediction with the first data collected at hospital patient admission and during the stay.

We implemented three different approaches. In the first approach (experiment 1), we included only available variables at admission. Experiment 2 evaluates the impact of additional laboratory data, the number of comorbidities, and the performance of the surgical intervention. These other variables are possible to be collected during the hospital stay. In Experiment 3, we tested the inclusion of more specific pathology-related variables, such as body mass index, symptoms, suggestive time of onset of Acute Coronary Syndrome (ACS Time), heart rate, and the number of segments with injury.

## Methods

**Fig. 1:**
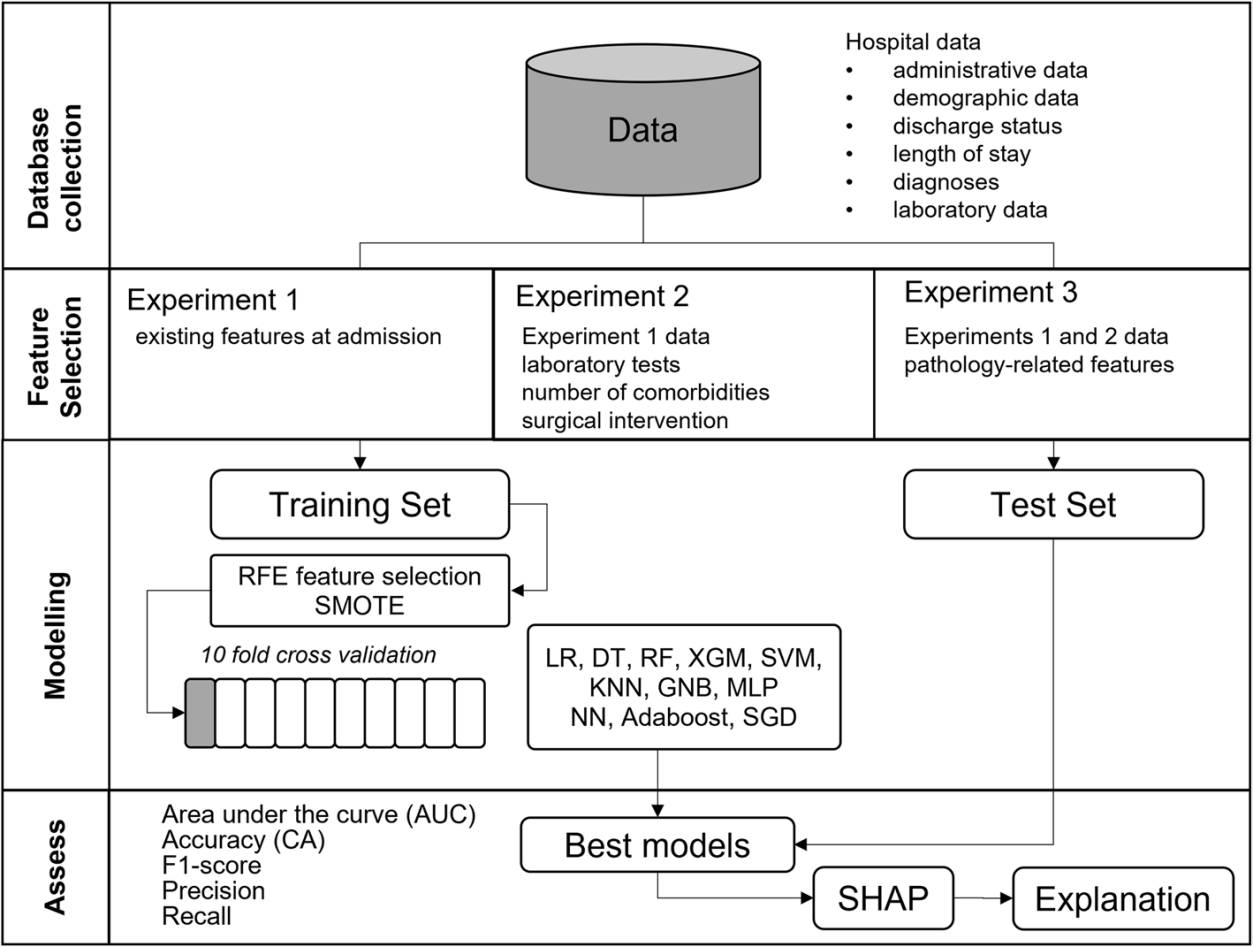
Process flow diagram of the proposed methodology

Figure 1 presents the proposed methodology with the following steps: (1) Database collection; (2) Feature Selection; (3) Modelling; and (4) Assessment of predictive capability, which are detailed in the following sections. The project was developed in Python using the following packages scikit-learn, pandas, numpy, imblearn.over_sampling and shap.

### Study design and study population

This is a cross-sectional and analytical observational retrospective study. The study population included patients aged 18 years or older with an episode of hospital discharge, where the primary diagnosis was AMI. Episodes from patients transferred to another hospital were excluded. The International Classification of Diseases, Ninth Version, Clinical Modification (ICD-9-CM) codes were used to identify AMI 410 – Acute Myocardial Infarction episodes except for subsequent episodes representing a sample of 1,761 episodes.

From this set of episodes, we excluded all the patients with a nonspecific AMI type, as the database contained only deceased patients. From this, we obtained a final sample of 1,749 episodes, representing 7.18% of the total inpatient episodes discharged with circulatory system pathology (24,359 episodes).

### Outcome

The outcome variable is in-hospital mortality, which assumes the value 0 for episodes whose outcome is “alive” and 1 for episodes whose outcome is “deceased”.

### Data collection

We used discharge data from a National Health Service (NHS) large hospital in Portugal (~ 1,000 beds) from 2013 to 2015. The anonymised database includes administrative data, demographic data, discharge status (alive or deceased), length of stay, diagnoses and procedures (ICD-9-CM) of episodes whose primary diagnosis was AMI, laboratory data (LD), and cardiac and physiologic test results.

### Feature selection and feature importance 

Clinical discussions led to the removal of unnecessary features from the dataset. Then, we performed feature selection through Recursive Feature Elimination (RFE) to improve the performance of the machine learning models [30]. The primary idea behind RFE is to create a model using all features, then select and eliminate the weakest feature, and repeat the process for the remaining features until a specified number of features is reached. Later, we used the SHAP library to interpret the models on our dataset. The SHAP framework is a comprehensive tool that has been designed to interpret the predictions made by machine learning models [31]. It represents a novel approach for explaining a wide range of black-box models and has been proven to be highly effective in terms of its interpretability performance [32].

### Pre-processing of data 

The missing values for fields where the percentage of missing was very low (less than 5%) were filled with the average or the most common value according to the variable [33]. This is a common approach in data analysis, especially when the percentage of missing values is low. On our case, the features Type of AMI, Age had less than 1% of missing values.

Due to some algorithms’ requirement on data to be in the same numerical range, we used the Min–Max normalisation for quantitative variables. Additionally, we used the One-Hot method to process the multi-category variables.

### Data imbalance

Skewed data is a challenging problem in clinical datasets, and it can adversely affect the performance of ML models. Therefore, we applied Synthetic Minority Oversampling Technique (SMOTE). SMOTE is a technique that creates synthetic data to oversample the minority classes in a dataset [33].

### Experimental setting

Three distinct experiments were created, varying in the independent variables used, as shown in Table 1. The objective was to test if introducing cardiac test results and physiological results to administrative data improved the model performance and to test if different methods to determine which classification model performs better.

**Table 1:** Independent variables under study

Variable	Experiment 1	Experiment 2	Experiment 3
Age	X	X	X
Sex	X	X	X
Type of AMI	X	X	X
Comorbidities	X	X	X
Laboratory findings (n)	25	33	33
Laboratory findings	Albumin, Erythrocyte Distribution Range (RDW-CV), Calcium, Creatinine, Creatine kinase (CK), Eosinophils, Erythrocytes, Glucose, Hematocrit, Haemoglobin, Mean globular haemoglobin (HGM), International Normalised ratio (INR), Lactate Dehydrogenase (LDH), Lymphocytes, Neutrophils, Platelets, Potassium, C-reactive protein, Sodium, Activated Partial Thromboplastin Time (APTT), Prothrombin time, Glutamic-oxalacetic transaminase, Glutamic-pyruvic transaminase (SGPT), Troponin I, Urea		
		Chlorine, Phosphokinase MM fraction (CK-MB), Arterial bicarbonate concentration (HCO3a), Mean corpuscular haemoglobin concentration (MCHC), Magnesium, Mean Platelet Volume (MPV), Blood oxygen (pO2), Blood oxygen saturation (sO2)	
N. º of comorbidities		X	X
Surgical Intervention		X	X
Body mass index			X
Symptoms			X
ACS Time			X
Heart Rate			X
N.º of Segments with Injury			X

For experiments 1 and 2, a dataset with 1,749 episodes was used. Regarding experiment 3, the number of episodes with detailed information on cardiac test results and physiological results corresponds to 445 episodes (25.4% of the 1,749 episodes).

Experiment 1 includes variables existing at admission time, and experiment 2 has more variables (eight additional laboratory findings, the number of comorbidities, and the performance of surgical intervention). These other variables are possible to collect during the hospital stay. In experiment 3, we tested the inclusion of more specific pathology-related variables.

Administrative and laboratory data were selected as independent variables (see Table 1). We segregated the laboratory findings into below-normal, normal, and above-normal levels. The relevant comorbidities for AMI selected were Anaemia, Cancer, Cardiogenic Shock, Diabetes with complications, Diabetes without complications, Cardiac dysrhythmia, Cerebrovascular Disease, Pulmonary edema, Acute Kidney Failure, Chronic Kidney Failure, and Respiratory infection, and have been selected according to the literature [34, 35]. The number of comorbidities represents the sum of one or more secondary diagnoses unrelated to each episode’s principal diagnosis (see Table 3 for more details).

The laboratory data included in all three experiments were Albumin, Erythrocyte Distribution Range (RDW-CV), Calcium, Creatinine, Creatine kinase (CK), Eosinophils, Erythrocytes, Glucose, Hematocrit, Haemoglobin, Mean globular haemoglobin (HGM), International Normalised ratio (INR), Lactate Dehydrogenase (LDH), Lymphocytes, Neutrophils, Platelets, Potassium, C-reactive protein, Sodium, Activated Partial Thromboplastin Time (APTT), Prothrombin time, Glutamic-oxalacetic transaminase, Glutamic-pyruvic transaminase (SGPT), Troponin I and Urea.

The following laboratory findings were used specifically for Experiments 2 and 3: Chlorine, Phosphokinase MM fraction (CK-MB), Arterial bicarbonate concentration (HCO3a), Mean corpuscular haemoglobin concentration (MCHC), Magnesium, Mean Platelet Volume (MPV), Blood oxygen (pO2) and Blood oxygen saturation (sO2).

#### Separation of data into training and test datasets

We used 70% of the dataset as training data, while 30% was allocated as testing to build the classifiers. We then used a tenfold cross-validation technique on the training set to avoid model overfitting and for hyperparameter tuning. The dataset was randomly divided into ten equal folds, each with approximately the same number of episodes; 10 validation experiments were performed, each used in turn as the validation set and the remaining nine used as the training set. We then used the 30% testing set to evaluate the model performance [36].

#### Predictive models

The dependent variable (hospital mortality) is categorical, which poses a classification problem. To mitigate this issue, we tested ten supervised learning methods, ranging from logistic regression to ensemble methods and neural networks.

- Logistic Regression [37];
- Decision Tree [38]
- Random Forest (RF) [39]
- Gradient Boosting [40]
- Support Vector Machine (SVM) [41];
- k-nearest neighbors (kNN) [42];
- Gaussian Naive Bayes [43];
- MLP Neural Network [41];
- AdaBoost [44];
- Stochastic Gradient Descent (SGD) [45].

A Grid Search method with a ten-fold CV was used to optimise the hyper-parameters of ML. Finally, the performance of each model was evaluated and compared in the test set. Table 2 presents the best hyperparameters used in this study for each method.

**Table 2:** Hyperparameters used for each learning method

Method	Hyperparameters
Logistic Regression	C: 0.1, penalty: L2, solver: liblinear
Decision Tree	criterion: entropy, max_depth: 5, min_samples_split: 10
Random Forest	criterion: entropy, max_depth: 10, min_samples_split: 2, n_estimators: 100, min_samples_leaf = 1, bootstrap = True, class_weight = None, ccp_alpha = 0.0
Gradient Boosting	learning_rate: 0.01, max_depth: 5, n_estimators: 300
SVM	C: 0.1, coef0: 0, degree: 2, gamma: scale, kernel: rbf, tol: 0.0001
k-NN	Number of neighbours: 7, Metric: minkowski, Weight:Uniform, leaf_size = 30, weights = ‘uniform’
MLP	activation: relu, alpha: 0.001, hidden_layer_sizes: (100,), solver: adam
Adaboost	n_estimators: 10, base_estimator = None, learning_rate = 1.0, algorithm = SAMME.R, random_state = None
Stochastic Gradient Descent	alpha: 0.001, penalty: elasticnet, epsilon = 0.1 l1_ratio = 0.15, learning_rate = ‘optimal’, loss = ‘hinge’, max_iter = 1000, n_iter_no_change = 5, penalty = ‘l2’, power_t = 0.5, tol = 0.001

#### Models’ evaluation

Measuring the success of machine learning algorithms is essential in determining their suitability. Classification performance can be measured in many ways: absolute ability, performance relative to other factors, probability of success, and others [13]. This paper uses the area under the curve (AUC), Classification Accuracy (CA), F1-score, Precision, and Recall.

## Results

### Descriptive statistics study population

Table 3 presents the descriptive statistics of the study population regarding Experiments 1 and 2. Of the 1,749 episodes in the study, 218 correspond to patients that died, corresponding to a mortality rate of 12.5%. Most patients were male (65.4%) and 70 years or older (51.8%). However, a higher mortality rate was observed in females (15.0%).

**Table 3:** Descriptive statistics of the study population – Experiments 1 and 2

		**Overall Study Sample**	**Mortality**
**Total**		1,749	218 (12.5%)
**Age Group**	< 70	843 (48.2%)	48 (5.7%)
	≥ 70	906 (51.8%)	170 (18.8%)
**Sex**	Female	606 (34.6%)	91 (15.0%)
	Male	1,143 (65.4%)	127 (11.1%)
**Type of AMI**	Anterior STEMI	300 (17.2%)	44 (14.7%)
	Other STEMI	785 (44.9%)	123 (15.7%)
	NSTEMI	664 (38.0%)	51 (7.7%)
**N.º of Comorbidities**	0	560 (32.0%)	17 (3.0%)
	1	470 (26.9%)	51 (10.9%)
	2	312 (17.8%)	57 (18.3%)
	3	189 (10.8%)	41 (21.7%)
	4	123 (7.0%)	33 (26.8%)
	5	67 (3.8%)	11 (16.4%)
	6	22 (1.3%)	5 (22.7%)
	7	5 (0.3%)	2 (40.0%)
	8	1 (0.1%)	1 (100.0%)
**Comorbidities**	Anaemia	220 (12.6%)	29 (13.2%)
	Cancer	88 (5.0%)	25 (18.4%)
	Cardiogenic Shock	105 (6.0%)	74 (70.5%)
	Diabetes with complications	150 (8.6%)	21 (14.0%)
	Diabetes without complications	395 (22.6%)	53 (13.4%)
	Cardiac dysrhythmia	481 (27.5%)	102 (21.2%)
	Cerebrovascular Disease	204 (11.7%)	31 (15.2%)
	Pulmonary oedema	96 (5.5%)	28 (29.2%)
	Acute Kidney Failure	359 (20.5%)	66 (18.1%)
	Chronic Kidney Failure	356 (20.4%)	54 (15.2%)
	Respiratory infection	209 (11.9%)	45 (21.5%)
**Surgical Intervention**	Yes	1,077 (61.6%)	93 (8.6%)
	No	672 (38.4%)	125 (18.6%)
**Number of tranfers between services**	< = 2	1472(84.2%)	180(12.2%)
	< = 5	248(14.2%)	24(9.7%)
	< = 8	26(1.5%)	4(15.4%)
	> 8	3(0.2%)	1(33.3%)

Of the three types of AMI analysed, other ST-Elevation Myocardial Infarction (STEMI) presented the highest prevalence (44.9%) and mortality rate (15.7%). Although Non-ST-Elevation Myocardial Infarction (NSTEMI) gave the second higher prevalence (38.0%), it showed the lowest mortality rate (7.7%).

Regarding the number of comorbidities, 68% of the episodes had at least one comorbidity at admission. Patients without comorbidity (*n* = 560) registered a lower mortality rate (3.0%). Cardiac dysrhythmia was the most frequent comorbidity (27.5%) and presented a mortality rate of 21.2%, followed by diabetes without complications, observed in 395 patients (22.6%). Cardiogenic Shock was observed in 105 patients, accounting for the highest mortality rate (70.5%).

Most of the patients had a surgical intervention (61.6%). Patients with surgical intervention presented a lower mortality rate (8.6%) than those without surgical intervention (18.6%).

Table 4 presents the characterisation of the study population’s laboratory findings, which were divided into below-normal, normal, and above-normal levels. Most patients present above-level results for Troponin I (82.6%), Neutrophils (78.9%), Lymphocytes (77.4%), C-reactive protein (56.3%), Creatinine (55.8%), Lactate Dehydrogenase (LDH) (52.1%) and Eosinophils (51.8%).

**Table 4:** Descriptive statistics of laboratory findings

Analytical results	Below level		Normal level		Above level	
	**Overall sample**	**Mortality**	**Overall sample**	**Mortality**	**Overall sample**	**Mortality**
Albumin	119(6.8%)	31(26.1%)	1,627(93.0%)	187(11.5%)	3(0.2%)	0(0.0%)
Erythrocyte Distribution Range (RDW-CV)	0(0.0%)	0(0.0%)	1,230(70.3%)	145(11.8%)	519(29.7%)	73(14.1%)
Calcium	200(11.4%)	30(15.0%)	1,543(88.2%)	188(12.2%)	6(0.3%)	0(0.0%)
Chlorine	58(3.3%)	7(12.1%)	1,501(85.8%)	200(13.3%)	190(10.9%)	11(5.8%)
Arterial bicarbonate concentration (HCO3a)	29(1.7%)	12(41.4%)	1,704(97.4%)	206(12.1%)	16(0.9%)	0(0.0%)
Mean corpuscular haemoglobin concentration (MCHC)	60(3.4%)	13(21.7%)	1,647(94.2%)	203(12.3%)	42(2.4%)	2(4.8%)
Creatinine	106(6.1%)	5(4.7%)	668(38.2%)	113(16.9%)	975(55.8%)	100(10.3%)
Phosphokinase MM fraction (CK-MB)	0(0.0%)	0(0.0%)	1,590(90.9%)	206(13.0%)	159(9.1%)	12(7.6%)
Creatine kinase (CK)	11(0.6%)	2(18.2%)	1,210(69.2%)	159(13.1%)	528(30.2%)	57(10.8%)
Eosinophils	0(0.0%)	0(0.0%)	843(48.2%)	177(21.0%)	906(51.8%)	41(4.5%)
Erythrocytes	650(37.2%)	80(12.3%)	1,086(62.1%)	135(12.4%)	13(0.7%)	5(38.5%)
Glucose	0(0.0%)	0(0.0%)	1,143(65.4%)	145(12.7%)	590(33.7%)	71(12.0%)
Haematocrit	670(38.3%)	80(11.9%)	1,052(60.2%)	133(12.6%)	27(1.5%)	5(18.5%)
Haemoglobin	559(32.0%)	75(13.4%)	1,177(67.3%)	141(12.0%)	13(0.7%)	2(15.4%)
Mean globular haemoglobin (HGM)	80(4.6%)	12(15.0%)	1,563(89.4%)	197(12.6%)	105(6.1%)	9(8.5%)
International Normalised ratio (INR)	0(0.0%)	0(0.0%)	1,631(93.3%)	179(11.0%)	118(6.8%)	39(33.1%)
Lactate Dehydrogenase (LDH)	3(0.2%)	0(0.0%)	835(47.7%)	57(6.8%)	911(52.1%)	98(10.8%)
Lymphocytes	1(0.1%)	1(100.0%)	394(22.5%)	97(24.6%)	1,354(77.4%)	120(8.9%)
Magnesium	6(0.3%)	0(0.0%)	1,738(99.4%)	217(12.5%)	5(0.3%)	1(20.0%)
Neutrophils	1(0.1%)	1(100.0%)	368(21%)	89(24.2%)	1,380(78.9%)	128(9.3%)
Blood oxygen (pO2)	45(2.6%)	8(17.8%)	1,633(93.4%)	198(12.1%)	71(4.1%)	12(16.9%)
Platelets	171(9.8%)	25(14.6%)	1,551(88.7%)	190(12.3%)	27(1.5%)	3(11.1%)
Potassium	74(4.2%)	8(10.8%)	1,570(89.8%)	187(11.9%)	105(6.0%)	23(21.9%)
C-reactive protein	0(0.0%)	0(0.0%)	764(43.7%)	110(14.4%)	985(56.3%)	108(11.0%)
Blood oxygen saturation (sO2)	17(1.0%)	6(35.3%)	1,717(98.2%)	209(12.2%)	15(0.9%)	3(20.0%)
Sodium	113(6.5%)	13(11.7%)	1,604(91.7%)	188(11.7%)	32(1.8%)	11(34.4%)
Activated Partial Thromboplastin Time (APTT)	14(0.8%)	5(35.7%)	1,486(85%)	176(11.8%)	249(14.2%)	37(14.9%)
Prothrombin time	1(0.1%)	0(0.0%)	1,549(88.6%)	166(10.7%)	199(11.4%)	52(26.1%)
Glutamic-oxalacetic transaminase	0(0.0%)	0(0.0%)	914(52.3%)	129(14.1%)	835(47.7%)	89(10.7%)
Glutamic-pyruvic transaminase (SGPT)	25(1.4%)	1(4.0%)	1,480(84.7%)	169(11.4%)	244(14.0%)	48(19.7%)
Troponin I	0(0.0%)	0(0.0%)	304(17.4%)	92(30.3%)	1,445(82.6%)	126(8.7%)
Urea	0(0.0%)	0(0.0%)	1,242(71%)	125(10.1%)	507(29.0%)	93(18.3%)
Mean Platelet Volume (MPV)	60(3.4%)	3(5.0%)	1,651(94.4%)	204(12.4%)	38(2.2%)	11(29.0%)

Regarding mortality, the highest rate was prominent in patients with results of HCO3a below level (41.4%), erythrocytes above level (38.5%), activated partial thromboplastin time (APTT) below level (35.7%), blood oxygen saturation (sO2) below level (35.3%), sodium above level (34.4%) and International Normalized Ratio (INR) above level (33.1%).

For Experiment 3, which included 445 episodes, Table 5 presents the study population characterisation. Of the 445 episodes, 32 correspond to patients that died, with a mortality rate of 7.2%. Most patients were male (76.0%) and less than 70 years old (69.0%). However, it was observed a higher mortality rate in women (9.3%) when compared with men (6.5%) and in patients 70 years or older (18.1%).

**Table 5:** Descriptive statistics of the study population – Experiment 3

		**Overall study sample**	**Mortality**
**Total**		445	32 (7.2%)
**Age Group**	< 70	307 (69.0%)	7 (2.3%)
	≥ 70	138 (31.0%)	25 (18.1%)
**Sex**	Female	107 (24.0%)	10 (9.3%)
	Male	338 (76.0%)	22 (6.5%)
**BMI**	Excess (≥ 25)	278 (62.5%)	18 (6.5%)
	Not excess (< 25)	123 (27.6%)	8 (6.5%)
	NA	44 (9.9%)	6 (13.6%)
**Symptoms**	Asymptomatic	4 (0.9%)	0 (0.0%)
	Tiredness	1 (0.2%)	0 (0.0%)
	Dyspnea	5 (1.1%)	2 (40.0%)
	Chest pain	409 (91.9%)	26 (6.4%)
	Cardiac arrest/Sudden death aborted	6 (1.3%)	0 (0.0%)
	Syncope	6 (1.3%)	0 (0.0%)
	Dizziness	8 (1.8%)	3 (37.5%)
	NA	6 (1.3%)	1 (16.7%)
**ACS Time**	< 6 h	285 (64.0%)	17 (6.0%)
	6 h to 12 h	81 (18.2%)	7 (8.6%)
	12 h to 24 h	39 (8.8%)	3 (7.7%)
	< 7 days	25 (5.6%)	3 (12.0%)
	< 15 days	3 (0.7%)	0 (0.0%)
	Unknown	1 (0.2%)	0 (0.0%)
	NA	11 (2.5%)	2 (18.2%)
**Heart Rate**	AVB 2nd degree, Mobitz I	2 (0.4%)	1 (50.0%)
	AVB 2nd degree, Mobitz II	1 (0.2%)	0 (0.0%)
	AVB 3rd degree	3 (0.7%)	2 (66.7%)
	Atrial fibrillation	18 (4.0%)	4 (22.2%)
	Pacing	6 (1.3%)	0 (0.0%)
	Junctional rhythm	1 (0.2%)	1 (100.0%)
	Sinus rhythm	389 (87.4%)	24 (6.2%)
	NA	25 (5.6%)	0 (0.0%)
**N. º of Segments with Injury**	1	126 (28.3%)	5 (4.0%)
	2	107 (24.0%)	6 (5.6%)
	3	79 (17.8%)	4 (5.1%)
	4	51 (11.5%)	4 (7.8%)
	5	33 (7.4%)	3 (9.1%)
	6	24 (5.4%)	6 (25.0%)
	7	15 (3.4%)	2 (13.3%)
	8	5 (1.1%)	2 (40.0%)
	9	1 (0.2%)	0 (0.0%)
	10	4 (0.9%)	0 (0.0%)

Approximately 92% of the patients presented chest pain, with a mortality rate of 6.4%. A mortality rate of 100% for junctional rhythm was observed, and 66.7% for AVB 3rd degree.

### Machine learning models

#### Experiment 1

As shown in Table 6, when applying feature selection and SMOTE, GNB and SGD were the methods that performed better in the test dataset (area under the ROC curve) when compared to the other classification methods, obtaining a an AUC value equal to 79%.

**Table 6:** Performance results of methods on Experiment 1 (Train and Test Dataset)

		**Model**	**Test**				
			**CA**	**Prec**	**Rec**	**AUC**	**F1**
No Feature Selection	Unbalanced	LR	0,89	0,55	0,10	0,54	0,17
		DT	0,89	0,50	0,18	0,58	0,27
		RF	0,91	0,68	0,35	0,66	0,46
		XGM	0,89	0,57	0,27	0,62	0,36
		SVM	0,89	0,00	0,00	0,50	0,00
		KNN	0,90	0,62	0,22	0,60	0,32
		**GNB**	0,83	0,34	0,53	**0,70**	0,42
		MLP NN	0,90	0,63	0,32	0,65	0,42
		Adaboost	0,90	0,68	0,25	0,62	0,37
		SGD	0,90	0,68	0,32	0,65	0,43
	SMOTE	LR	0,81	0,34	0,72	0,77	0,46
		DT	0,84	0,39	0,68	0,77	0,50
		RF	0,90	0,60	0,47	0,71	0,52
		XGM	0,90	0,57	0,53	0,74	0,55
		SVM	0,88	0,49	0,52	0,72	0,50
		KNN	0,78	0,31	0,75	0,77	0,44
		**GNB**	0,81	0,35	0,77	**0,79**	0,48
		MLP NN	0,86	0,42	0,52	0,71	0,47
		Adaboost	0,89	0,53	0,45	0,70	0,49
		SGD	0,77	0,31	0,82	0,79	0,45
With Feature Selection	Unbalanced	LR	0,90	0,61	0,28	0,63	0,39
		DT	0,88	0,43	0,17	0,57	0,24
		RF	0,87	0,41	0,28	0,62	0,34
		XGM	0,89	0,52	0,18	0,58	0,27
		SVM	0,89	0,50	0,05	0,52	0,09
		KNN	0,90	0,61	0,28	0,63	0,39
		**GNB**	0,87	0,44	0,47	**0,69**	0,45
		MLP NN	0,90	0,60	0,25	0,61	0,35
		Adaboost	0,89	0,60	0,20	0,59	0,30
		SGD	0,90	0,65	0,33	0,65	0,44
	SMOTE	LR	0,80	0,33	0,73	0,77	0,46
		DT	0,85	0,36	0,47	0,68	0,41
		RF	0,86	0,39	0,38	0,65	0,39
		XGM	0,87	0,44	0,58	0,74	0,50
		SVM	0,84	0,39	0,68	0,77	0,49
		KNN	0,82	0,34	0,62	0,73	0,44
		**GNB**	0,82	0,37	0,75	**0,79**	0,49
		MLP NN	0,82	0,34	0,65	0,74	0,45
		Adaboost	0,83	0,37	0,62	0,74	0,46
		**SGD**	0,80	0,34	0,77	**0,79**	0,47

Regarding the remaining metrics: CA, precision, recall and F1-score, GNB obtained 82%, 37%, 75% and 49%, respectively. At the same time, SGD obtained 80%, 34%, 77% and 47%, respectively, on the test dataset.

#### Experiment 2

In Experiment 2, the same variables from Experiment 1 were used, and eight laboratory findings were added, as well as the comorbidities number and the performance of the surgical intervention. Table 7 presents the performance obtained for the ten learning methods tested.

**Table 7:** Performance results of methods on Experiment 2 (Train and Test Dataset)

		**Model**	**Test**				
			**CA**	**Prec**	**Rec**	**AUC**	**F1**
No Feature Selection	Unbalanced	LR	0,89	0,58	0,12	0,55	0,19
		DT	0,92	0,64	0,62	0,79	0,63
		RF	0,93	0,80	0,47	0,73	0,59
		XGM	0,91	0,68	0,45	**0,71**	0,54
		SVM	0,89	0,00	0,00	0,50	0,00
		KNN	0,90	0,65	0,28	0,63	0,40
		GNB	0,83	0,35	0,57	0,72	0,44
		MLP NN	0,90	0,64	0,38	0,68	0,48
		Adaboost	0,90	0,68	0,25	0,62	0,37
		SGD	0,90	0,58	0,32	0,64	0,41
	SMOTE	LR	0,84	0,39	0,77	**0,81**	0,52
		DT	0,89	0,52	0,67	0,79	0,58
		RF	0,93	0,80	0,47	0,73	0,59
		XGM	0,92	0,69	0,57	0,77	0,62
		SVM	0,89	0,50	0,48	0,71	0,49
		KNN	0,78	0,31	0,77	0,77	0,44
		GNB	0,80	0,33	0,72	0,76	0,45
		MLP NN	0,88	0,48	0,53	0,73	0,51
		Adaboost	0,90	0,57	0,50	0,73	0,53
		SGD	0,56	0,19	0,90	0,71	0,32
With Feature Selection	Unbalanced	LR	0,90	0,63	0,28	0,63	0,39
		DT	0,87	0,38	0,18	0,57	0,25
		RF	0,90	0,58	0,43	**0,70**	0,50
		XGM	0,89	0,59	0,22	0,60	0,32
		SVM	0,89	0,67	0,13	0,56	0,22
		KNN	0,90	0,66	0,35	0,66	0,46
		GNB	0,88	0,46	0,38	0,66	0,42
		MLP NN	0,90	0,61	0,23	0,61	0,34
		Adaboost	0,90	0,68	0,22	0,60	0,33
		SGD	0,90	0,68	0,32	0,65	0,43
	SMOTE	LR	0,81	0,34	0,75	0,78	0,47
		DT	0,87	0,46	0,62	0,76	0,52
		RF	0,88	0,47	0,48	0,71	0,48
		XGM	0,89	0,51	0,60	0,76	0,55
		SVM	0,85	0,42	0,75	0,81	0,54
		KNN	0,83	0,35	0,57	0,71	0,43
		GNB	0,85	0,40	0,70	0,78	0,51
		MLP NN	0,82	0,35	0,72	0,77	0,47
		Adaboost	0,86	0,43	0,62	0,76	0,51
		SGD	0,72	0,27	0,83	0,77	0,41

When applying feature selection and SMOTE, SVM was the method with the best performance in the test dataset regarding the AUC (81%), followed by the LR and GNB methods (78%).

#### Experiment 3

For Experiment 3, in addition to those used in the previous experiments, the set of new morbidity variables and test results were used. Table 8 presents the performance results.

**Table 8:** Performance results of methods on Experiment 3 (Train and Test Dataset)

		**Model**	**Test**				
			**CA**	**Prec**	**Rec**	**AUC**	**F1**
No Feature Selection	Unbalanced	LR	0,95	0,67	0,60	0,79	0,63
		DT	0,96	0,70	0,70	**0,84**	0,70
		RF	0,92	0,33	0,10	0,54	0,15
		XGM	0,92	0,40	0,20	0,59	0,27
		SVM	0,93	0,50	0,60	0,78	0,55
		KNN	0,93	0,00	0,00	0,50	0,00
		GNB	0,13	0,07	0,90	0,49	0,13
		MLP NN	0,94	0,60	0,60	0,78	0,60
		Adaboost	0,88	0,13	0,10	0,52	0,11
		SGD	0,93	0,55	0,60	0,78	0,57
	SMOTE	LR	0,93	0,53	0,80	0,87	0,64
		DT	0,90	0,36	0,40	0,67	0,38
		RF	0,92	0,33	0,10	0,54	0,15
		XGM	0,90	0,36	0,40	0,67	0,38
		SVM	0,94	0,63	0,50	0,74	0,56
		KNN	0,87	0,36	0,90	**0,89**	0,51
		GNB	0,18	0,08	0,90	0,51	0,14
		MLP NN	0,95	0,62	0,80	0,88	0,70
		Adaboost	0,89	0,33	0,50	0,71	0,40
		SGD	0,94	0,60	0,60	0,78	0,60
With Feature Selection	Unbalanced	LR	0,94	0,60	0,60	0,78	0,60
		DT	0,96	0,70	0,70	0,84	0,70
		RF	0,93	0,60	0,30	0,64	0,40
		XGM	0,92	0,33	0,10	0,54	0,15
		SVM	0,96	0,67	0,80	**0,88**	0,73
		KNN	0,93	0,50	0,10	0,55	0,17
		GNB	0,93	0,50	0,80	0,87	0,62
		MLP NN	0,93	0,50	0,40	0,68	0,44
		Adaboost	0,91	0,40	0,40	0,68	0,40
		SGD	0,93	0,60	0,30	0,64	0,40
	SMOTE	LR	0,91	0,44	0,80	0,86	0,57
		DT	0,89	0,33	0,50	0,71	0,40
		RF	0,92	0,40	0,20	0,59	0,27
		XGM	0,95	0,71	0,50	0,74	0,59
		SVM	0,93	0,50	0,20	0,59	0,29
		KNN	0,91	0,44	0,70	0,81	0,54
		GNB	0,88	0,35	0,70	0,80	0,47
		MLP NN	0,92	0,45	0,50	0,73	0,48
		Adaboost	0,93	0,50	0,50	0,73	0,50
		SGD	0,94	0,57	0,80	**0,88**	0,67

When applying feature selection and SMOTE, SGD was the method with the best performance on the test dataset regarding the AUC metric (88%), followed by LR (86%). KNN and GNB obtained an AUC equal to or above 80%.

### Feature importance in the experiments

To understand how variables impact the model’s output on each experiment proposed, we used SHAP on the best predictive model achieved for each experiment. Table 9 summarises the results of the experiments created with the best performances.

**Table 9:** Summary of the results of the experiments created with the best performances

Experiment	FS	Oversampling	Method	CA	Prec	Rec	AUC	F1
1	Yes	Yes	SGD	0.80	0.34	0.77	0.79	0.47
2	Yes	Yes	SVM	0.85	0.42	0.75	0.81	0.54
3	No	Yes	KNN	0.87	0.36	0.90	0.89	0.51

**Fig. 2:**
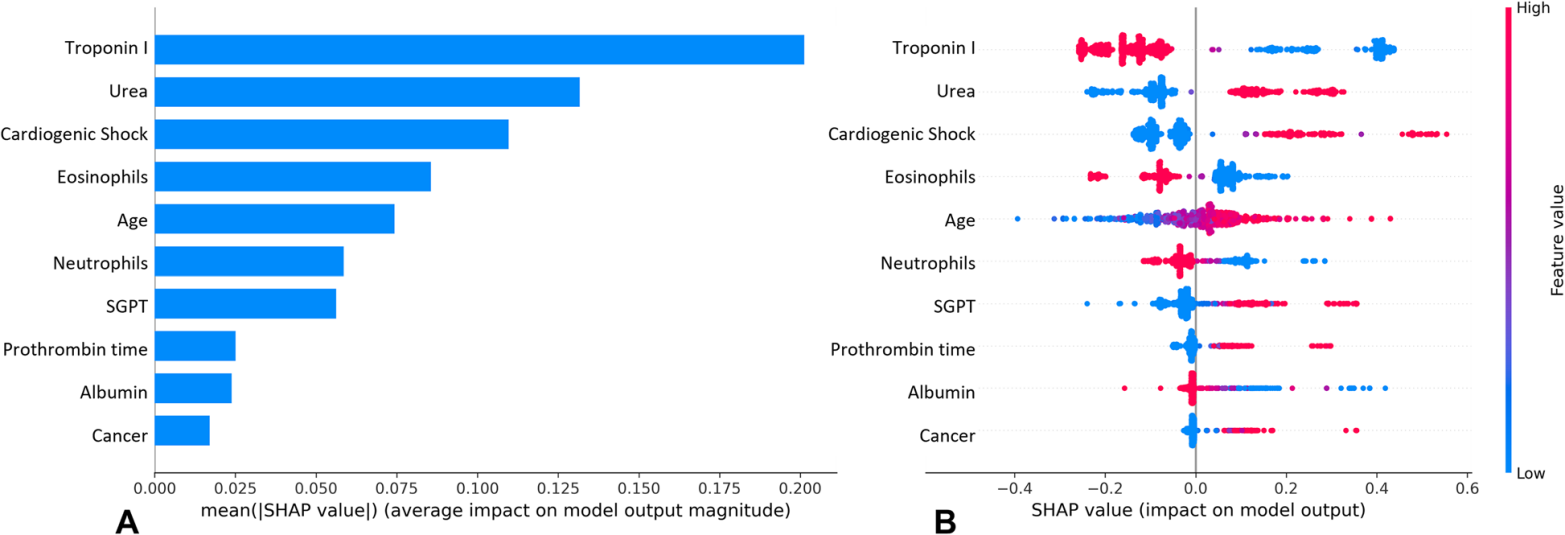
Experiment 1 model interpretation. **A** The importance ranking of the top 10 variables according to the mean (|SHAP value|); **B** The importance ranking of the top 10 risk factors with stability and interpretation using the optimal model

**Fig. 3:**
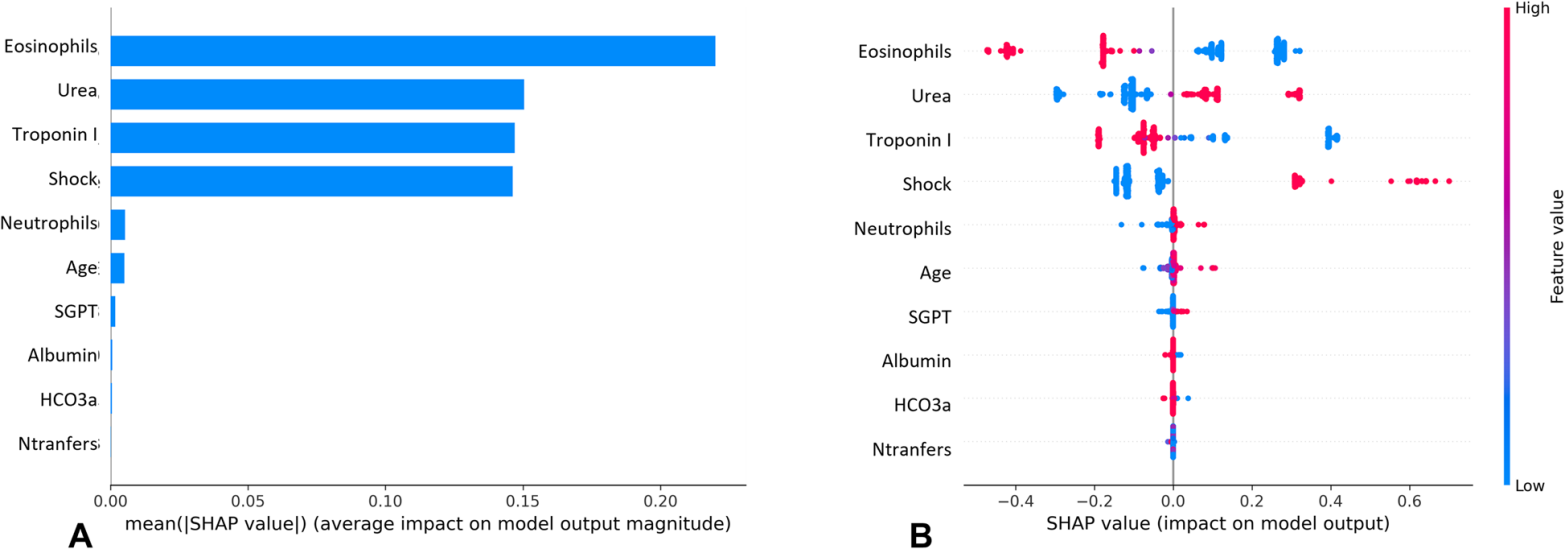
Experiment 2 model interpretation. **A** The importance ranking of the top 10 variables according to the mean (|SHAP value|); **B** The importance ranking of the top 10 risk factors with stability and interpretation using the optimal model

**Fig. 4:**
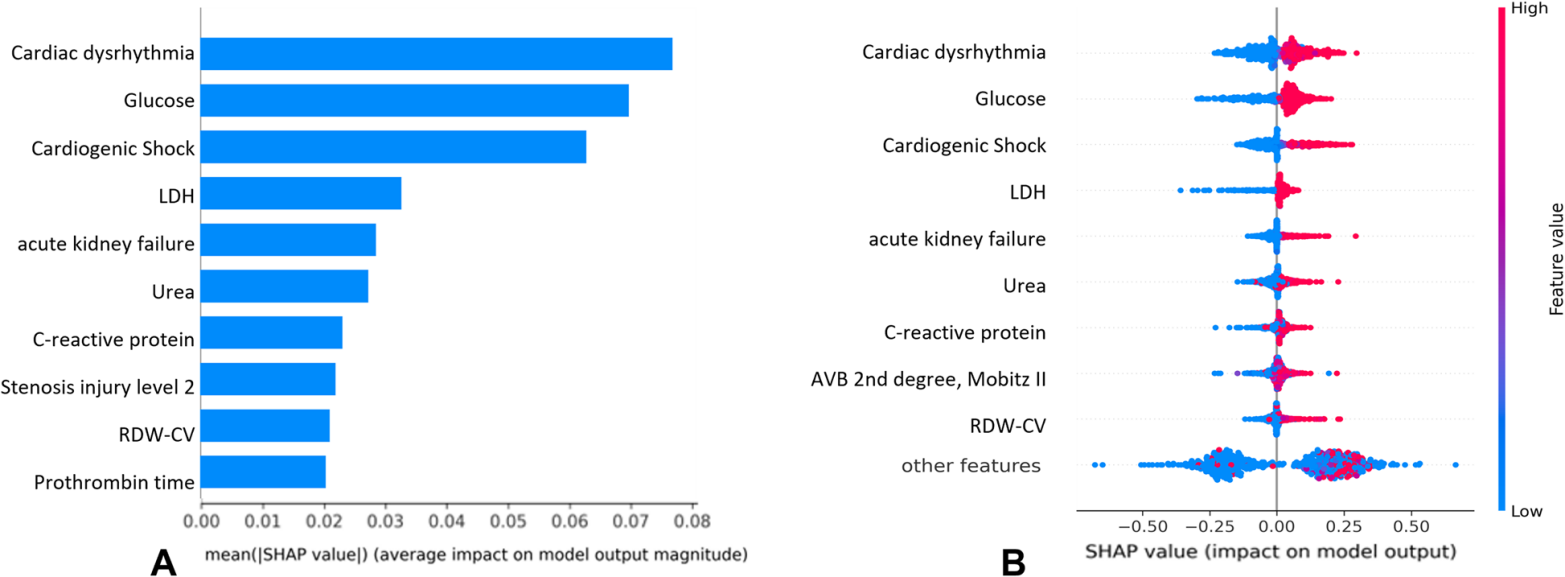
Experiment 3 model interpretation. **A** The importance ranking of the top 20 variables according to the mean (|SHAP value|); **B** The importance ranking of the top 10 risk factors with stability and interpretation using the optimal model

The top 10 risk factors were evaluated by their average absolute SHAP value and can be seen in Figs. 2A, 3A and 4A (for each experiment). Additionally, Figs. 2B, 3B and 4B displays the top 10 most important features for each experiment’s best model, with the y-axis indicating the importance of the predictive model and the x-axis representing the unified index that responds to the influence of a particular feature in the model. Each important feature row depicts the attribution of all patients to the outcome using dots of different colours, with red dots indicating high-risk values and blue dots representing low-risk values.

Regarding our first experiment, a higher value on urea, cardiogenic shock, older age, SGPT, prothrombin time and cancer were associated with higher predicted probability mortality. Furthermore, lower values of troponin I, eosinophils, neutrophils and albumin were found to be associated with a higher predicted probability of mortality.

For the second experiment, higher values of urea, cardiogenic shock, neutrophils, age and SGPT were found to be associated with a higher predicted probability of mortality, while lower values of eosinophils, troponin I, albumin and HCo3a also increased the risk of mortality.

Finally, for the third experiment, higher values on cardiac dysrhythmia, glucose, cardiogenic Shock, LDH, acute kidney failure, Urea, C-reactive protein, Nr of Segments with Injury 2, RDW-CV and prothrombin time were found to be associated with a higher predicted probability of mortality.

## Discussion

In this study, we analysed the use of ten supervised machine learning methods to predict AMI in-hospital mortality. The aim was to build experiments with different approaches to determine which classification model performs better and whether introducing cardiac test results and physiological results to administrative data improve the model performance.

Regarding Experiment 1, SGD presented the best performance, with an AUC of 79% and recall of 77%, applying feature selection and oversampling, while in Experiment 2, SVM presented the best performance, with an AUC of 81% and recall of 75%, also applying feature selection and oversampling.

Regarding Experiment 3, KNN performed best on the test dataset, with an AUC of 89% and a recall of 90%, only applying oversampling but not feature selection. However, when using both oversampling and feature selection, SGD performed best, with an AUC of 88% and a recall of 80%.

Therefore, in the same conditions (feature selection and oversampling), the models’ performance was observed, suggesting the relevance of including more specific variables, such as cardiac test results and physiological results.

The number of publications on predicting the mortality of AMI using machine learning is still limited, most of which are based on scoring scales and Logistic Regression models that tend to have lower performances. Nevertheless, recent literature that used laboratory findings and symptoms presents better discriminative performance using an ML-based approach than traditional risk-scoring methods such as TIMI [26, 27].

Specifical, Aziz et al. [27] model performance using a complete and reduced variable produced an area under the receiver operating characteristic curve (AUC) from 0.73 to 0.90. Overall ML model performed better than TIMI for in-hospital, 30 days and 1-year AUC (0.88 vs 0.81, 0.90 vs 0.80, 0.84 vs 0.76). Aziz et al. [27] study is comparable with the results found in the present study for Experiment 3. As well as Khera et al. [46] results, whose AUC was 89.8% for the XGBoost method and 89.9% for the meta-classifier.

The study’s results suggest that including new variables, mainly cardiac test results and physiological results, and complex interactions between them can increase the performance of predictions in this context since they help identify patients at risk and reduce false positives and negatives.

Experiment 3 also includes vital signs, such as pain and heart rate, similar to TIMI and GRACE [47].

In the three experiments of this study, cardiogenic Shock and Urea were in the top 10 variables associated with a higher probability of mortality. These results align with the literature once cardiogenic shock was identified as the most common cause of death in patients hospitalised with AMI [48–51]. Regarding Urea, according to Zhu et al. [52], blood urea nitrogen was robustly associated with increased short-term mortality in patients with Cardiogenic Shock after AMI; Horiuchi et al. [53] also found that blood urea nitrogen is a predictor of in-hospital mortality in AMI patients.

The other risk factors identified are consistent with the literature, with advanced age, cancer, cardiac dysrhythmia, prothrombin time, and eosinophils already been highlighted and explained previously [30]. Tu et al. [54] identified diabetes, cancer, and renal failure as predictors of AMI. Thus, several of these comorbidities at admission influence the risk of death [54, 55].

Previous studies have also shown that the value of neutrophils is higher in patients with complications in AMI. Thus, it was considered a strong and independent predictor of in-hospital mortality in patients with AMI and ST-segment elevation [56].

Although the contributions of this study reinforce the importance of applying a machine learning system to predict AMI mortality, it presents several limitations. Specifically, the small sample size, particularly regarding Experiment 3; the data originating from a single hospital; and the data period that refers to 2013–2015, which could potentially be outdated.

In addition to those limitations, the implementation of a Machine Learning approach to support health care poses some challenges crucial to overcome, such as:

- The time and cost associated with the collection and processing of data
- The lack of data and systems interoperability;
- The lack of trained professionals;
- The lack of allocated and dedicated human resources.

Further studies should be conducted and consider the inclusion of more variables that may be relevant in predicting AMI mortality, such as socioeconomic factors, systolic blood pressure, heart rate, and electrocardiogram results. We also suggest creating mortality prediction models for other stages of care, such as at discharge, using different variables to analyse their influence on mortality, such as length of stay and length of stay in the intensive care unit.

We also consider that there is potential to extend this research to other pathologies with high mortality, such as other circulatory system diseases, malignant neoplasms, and respiratory system conditions.

## Conclusions

Given the significant mortality rate of AMI, predicting its risk of death can assist healthcare organisations and their professionals in allocating the provision of care based on risk. Prediction models allow improved outcomes, based on more informed and accurate decision-making.

In conclusion, introducing new variables into the ML model impacts the performance of the methods, reinforcing the premise that no single approach is adapted to all situations but must be selected, taking into account the context and the information available.

All relevant variables identified in the different models are described in the literature as associated with a worse prognosis and a higher risk of death in AMI. Thus, similar to other studies in this area, this investigation demonstrates that the machine learning methods created could be valuable tools in clinical practice decision-making. Integrating machine learning can potentially transform care delivery and provide an increasingly accurate toolkit. When incorporated into information systems, they can make clinical practice more efficient, faster, personalised, and effective, reducing waste by optimising resources and installed capacity and improving the patient journey. In the era of Big Data, AI emerges as an alternative to traditional models since it can explore a large amount of information automatically and systematically with better performance, as proven in this study.

For such improvements to take place, there is a need to:

- Continue research and development of improved mortality prediction models for the pathologies with the most significant morbidity and mortality rates;
- Comprehend that optimal machine learning models only work if they have superior processes implemented around them;
- Invest in technological infrastructure, implement standards that allow data and systems interoperability, and create a single repository with all types of available data;
- Increase digital health literacy among professionals for a smooth digital transition in the healthcare industry;

## Data Availability

The data that supports the findings of this study is only available upon reasonable request to Teresa Magalhães (teresa.magalhaes@ensp.unl.pt) and with permission of Centro Hospitalar Universitário Lisboa Norte, E.P.E.
